# Multidimensional evaluation of the clinical efficacy and potential mechanisms of acupuncture combined with rehabilitation training in the treatment of stroke: a study based on multiple evaluation indicators

**DOI:** 10.3389/fneur.2025.1573073

**Published:** 2025-05-21

**Authors:** Jingjun Xie, Jinxia Li, Qi Sun, Jie Jiang

**Affiliations:** ^1^The First People's Hospital of Huzhou, Huzhou, Zhejiang, China; ^2^Huzhou Hospital of Traditional Chinese Medicine, Affiliated to Zhejiang Chinese Medical University, Huzhou, Zhejiang, China

**Keywords:** stroke, acupuncture, rehabilitation training, neurological function, activities of daily living ability

## Abstract

**Objective:**

This study aims to explore the therapeutic effects and potential mechanisms of acupuncture combined with rehabilitation training on stroke patients.

**Methods:**

A total of 120 stroke patients were randomly divided into a control group (Group A), a rehabilitation training group (Group B), and an acupuncture combined with rehabilitation training group (Group C), with 40 patients in each group. The National Institute of Health Stroke Scale (NIHSS) for neurological deficit, the Barthel Index for activities of daily living ability were evaluated before treatment, 4, 8, and 12 weeks after treatment. Additionally, the levels of serum brain-derived neurotrophic factor (BDNF), nerve growth factor (NGF), and inflammatory factors (IL-6, TNF-α) were detected.

**Results:**

The acupuncture combined with rehabilitation training group (Group C) was significantly superior to the other two groups (Group A and Group B) in improving neurological function, activities of daily living ability, and regulating serum factor levels.

**Conclusion:**

Acupuncture combined with rehabilitation training is an effective treatment regimen for stroke, providing a theoretical and practical basis for clinical application.

## Introduction

Stroke, as a leading cause of adult disability and mortality worldwide, imposes a profound socioeconomic burden due to its high incidence, recurrence rate, and long-term neurological sequelae 1. A comprehensive systematic analysis for the Global Burden of Disease Study 2019 revealed the staggering global prevalence of stroke, with an estimated 10.1 million new cases and 5.5 million deaths annually (1). In China, epidemiological studies have also highlighted the growing burden, with incidence rates ranging from 109.7 to 217 per 100,000 population. Approximately 75% of stroke survivors experience moderate-to-severe functional impairments in motor, cognitive, or language domains, significantly compromising their quality of life (2). While acute-phase interventions like thrombolysis and neuroprotection are critical, post-stroke rehabilitation remains the cornerstone for functional recovery. Conventional rehabilitation therapies, such as task-specific training and robotic-assisted therapy, have demonstrated efficacy in promoting neuroplasticity, yet their impact on deep-brain circuit reconstruction and systemic neural repair remains limited (3).

Acupuncture, an ancient therapeutic modality rooted in Traditional Chinese Medicine, has emerged as a promising adjunctive therapy for stroke rehabilitation. Its mechanism of action integrates neuroendocrine regulation, anti-inflammatory effects, and promotion of angiogenesis (4). Notably, multiple high-quality clinical studies have highlighted acupuncture's unique advantages in enhancing neural repair. For example, a multicenter randomized controlled trial (RCT) involving 154 stroke patients showed that acupuncture significantly improved neurological and motor function scores and reduced disability rates (5). A total of 24 randomized controlled studies included 2,133 patients with post-stroke dyskinesia were included. The total effective rate of acupuncture was more advantageous in the treatment of post-stroke dyskinesia (relative risk = 1.31, 95% confidence interval [CI] [1.22–1.42], *Z* = 6.96, *P* < 0.0001) (6). Mechanistic studies reveal that acupuncture may modulate multiple neural repair pathways. Animal models show that acupuncture stimulation activates the phosphatidylinositol 3-kinase (PI3K)/Akt signaling pathway, promoting neuronal survival and axonal sprouting in the ischemic brain (7). Neuroimaging studies have tentatively confirmed that acupuncture could promote neuroplasticity in cortical motor areas in stroke patients, which improves motor disorders (8). These findings collectively support acupuncture's role in bridging the gap between local neural repair and systemic functional recovery.

This study aims to systematically evaluate the clinical efficacy of acupuncture combined with rehabilitation training in the recovery of neurological function in stroke patients through a prospective randomized controlled design, and explore its mechanism of action from multiple targets such as neural plasticity and inflammation regulation, providing high-level evidence-based medical evidence for optimizing stroke rehabilitation programs.

## Materials and methods

### Study design

**Figure 1:**
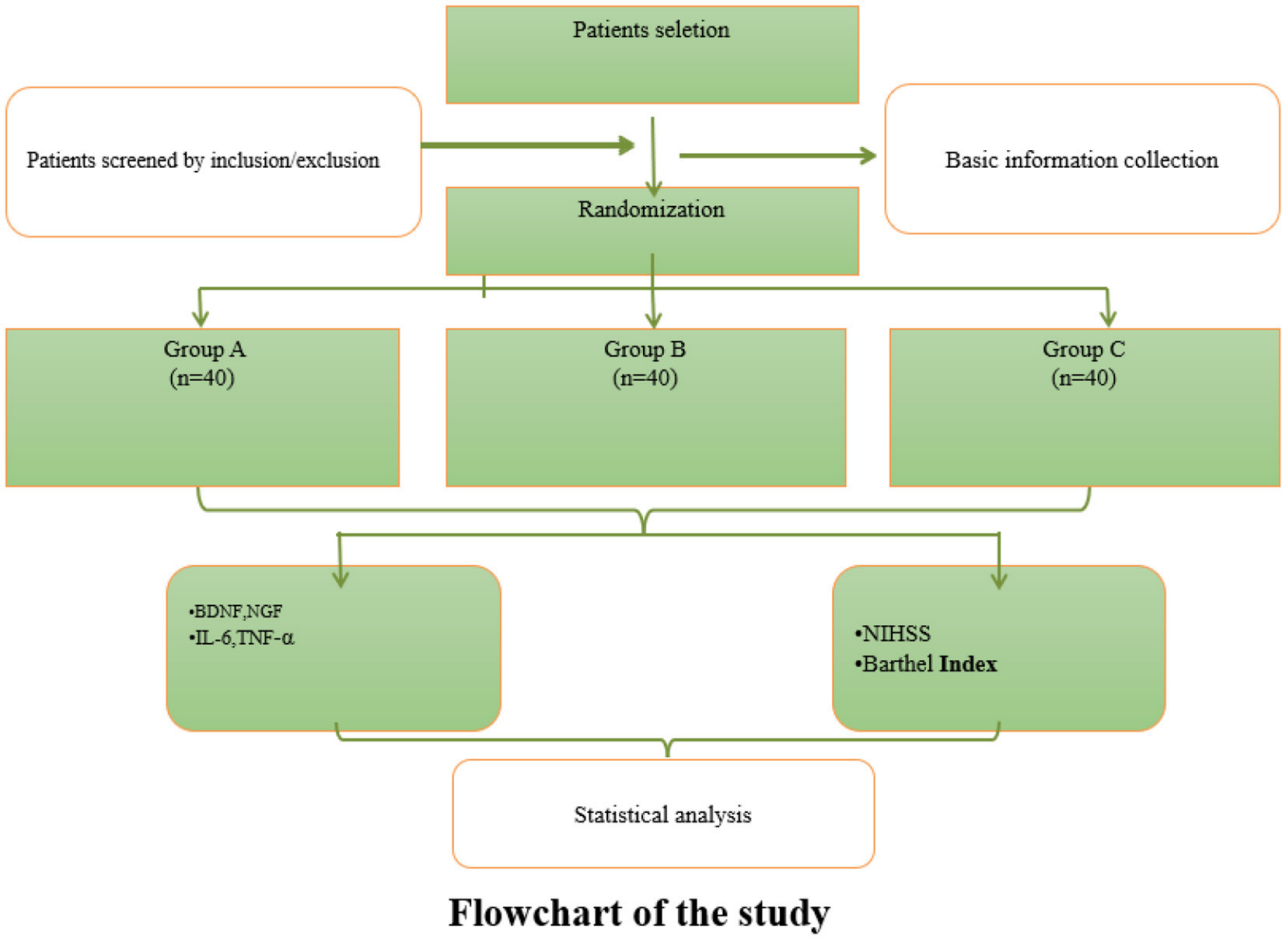
Flowchart of the study.

This study employed a prospective, randomized, single-blind, three-arm controlled design. A total of 120 eligible stroke patients were randomly allocated into three groups using a computer-generated random number table: Group A (control, routine treatment), Group B (rehabilitation training + routine treatment), and Group C (acupuncture + rehabilitation training + routine treatment). Interventions were administered for 12 weeks, with efficacy assessments conducted at baseline, 4, 8, and 12 weeks post-treatment. Blinding was applied to outcome assessors and statisticians to minimize bias. Figure 1 illustrates the flow of this study.

### Sample size calculation

The sample size was determined a priori using G^*^Power 3.1 software based on preliminary data from a pilot study (*n* = 30) and previous literature (9). Assuming an effect size (Cohen's d) of 0.5 (moderate effect), a two-tailed significance level (α) of 0.05, and a statistical power (1-β) of 0.8, the minimum required sample size was 36 participants per group. To account for a potential attrition rate of 10% (10), we enrolled 40 patients per group, resulting in a total of 120 participants. All 120 enrolled patients completed the 12-week trial without dropout or missing data. Compliance with treatment protocols was monitored weekly, and no participants withdrew due to adverse events or non-adherence. Data integrity was ensured by strict follow-up procedures, and all outcome assessments were fully recorded.

### Research subjects

A total of 120 stroke patients from The First People's Hospital of Huzhou from December 2023 to December 2024 were selected. **Inclusion criteria:** ① Meeting the diagnostic criteria for ischemic stroke revised by the Fourth National Conference on Cerebrovascular Diseases and confirmed by cranial CT or MRI; ② First onset, with a disease course within 30 days; ③ Aged 40–75 years old; ④ Patients and their families signed the informed consent form. **Exclusion criteria:** ① Complicated with severe organ dysfunctions such as heart, liver, and kidney; ② With a history of mental illness or cognitive impairment and unable to cooperate with treatment; ③ With contraindications to acupuncture. The patients were randomly divided into three groups according to the random number table, with 40 patients in each group. The control group received routine treatment, the rehabilitation training group received routine treatment plus rehabilitation training, and the acupuncture combined with rehabilitation training group received routine treatment plus rehabilitation training plus acupuncture. This study was approved by the hospital's medical ethics committee [Ethics Review No. (2023KYLL036)], and registered at the China Clinical Trial Registry(ChiCTR2300072096), all patients in the three groups signed the informed consent form.

### Treatment methods

#### Routine treatment

Patients were given drugs for antiplatelet aggregation, improvement of cerebral circulation, and nerve nutrition. The dosage and types of drugs were adjusted according to the specific conditions of the patients.

#### Rehabilitation training

Rehabilitation training was started 48 h after the patients' vital signs were stable.

The training content included:

##### Limb movement training

Bobath technique, Brunnstrom technique, etc. were used for joint range of motion training. For example, flexion, extension, and rotation activities were carried out on the shoulder, elbow, wrist, hip, knee, and ankle joints, with each joint moving 10–15 times, 2–3 groups per day; Muscle strength training was carried out through assisted movement, active movement, and resistance movement to enhance limb muscle strength. For example, elastic bands were used for upper limb extension and flexion training, with each training session lasting 15–20 min; Limb coordination training included fine finger movement training (such as buttoning, picking up beans, etc.) and lower limb alternating movement training, with each training session lasting 10–15 min.

##### Balance training

Static balance training was carried out with the help of equipment such as balance boards and balance balls. Patients were asked to sit on the balance board or balance ball and maintain body stability, with each training session lasting 10–15 min; Dynamic balance training, such as center of gravity transfer training on the balance board, was carried out, with each training session lasting 10–15 min.

##### Walking training

According to the patients' recovery situation, weight-bearing walking training was first carried out. Weight-bearing equipment was used to reduce the patients' body weight and help them practice walking, with each training session lasting 20–30 min; Then, gait training was carried out to correct abnormal gaits such as foot drop and circumduction gait, with each training session lasting 15–20 min.

##### Activities of daily living ability training

This included training in dressing, eating, washing, and using the toilet. During dressing training, patients were guided on the correct dressing sequence and method, with each training session lasting 10–15 min; During eating training, appropriate food and eating methods (such as semi-liquid food, small mouthful feeding, etc.) were selected according to the patients' swallowing function, with each training session lasting 15–20 min; During washing training, patients were helped to complete actions such as brushing teeth, washing face, and washing hands, with each training session lasting 10–15 min; During toilet training, patients were trained to independently complete the transfer from the wheelchair to the toilet and the excretion process, with each training session lasting 10–15 min. Each training session lasted 60 min, once a day, 5 times a week, for a total of 12 weeks.

#### Acupuncture treatment

Acupoints on the affected limb were selected, such as Jianyu (LI15), Quchi (LI11), Shousanli (LI10), Waiguan (SJ5), Hegu (LI4), Huantiao (GB30), Yanglingquan (GB34), Zusanli (ST36), Sanyinjiao (SP6), Taichong (LR3), etc. The acupoint locations were based on the standards in the “Acupuncture and Moxibustion” textbook. The even reinforcing-reducing method was used. After needle insertion to obtain qi, the needles were retained for 30 min, with needling manipulation performed 2–3 times during this period, each manipulation lasting 1–2 min. The manipulation method was lifting-thrusting and twirling, with the lifting-thrusting amplitude of 0.3–0.5 inches and the twirling angle of 180°-360°. Treatment was given once a day, 5 times a week, for 12 weeks.

### Observation indicators

#### Neurological deficit score (NIHSS)

The NIHSS was used to score the degree of neurological deficit in patients before treatment, 4, 8, and 12 weeks after treatment. This scale includes 11 items such as level of consciousness, gaze, visual field, facial paralysis, upper limb movement, lower limb movement, ataxia, sensation, language, dysarthria, and neglect. The higher the score, the more severe the neurological deficit.

#### Activities of daily living ability score (Barthel Index)

The Barthel Index was used to evaluate the patients' activities of daily living ability at the corresponding time points. It includes 10 items such as eating, bathing, grooming, dressing, bowel control, bladder control, toilet use, bed-chair transfer, walking on a flat surface, and going up and down stairs. The higher the score, the better the activities of daily living ability.

#### Serological index detection

Fasting venous blood samples were collected from patients in the early morning before treatment and 12 weeks after treatment. Serum was separated by centrifugation, and the levels of serum brain-derived neurotrophic factor (BDNF), nerve growth factor (NGF), and inflammatory factors (IL-6, TNF-α) were detected by enzyme-linked immunosorbent assay (ELISA). The operation was carried out strictly in accordance with the instructions of the kit.

### Statistical methods

SPSS 25.0 statistical software was used to analyze the data. Measurement data were expressed as mean ± standard deviation (x ± s). One-way ANOVA was used for comparison among multiple groups, and repeated-measures ANOVA was used for comparison at different time points within the group. The LSD – *t*-test was used for pairwise comparisons. A *P* < 0.05 was considered statistically significant.

## Results

### Comparison of general data of the three groups of patients

There were no statistically significant differences in age, gender, disease course, baseline NIHSS score, and Barthel Index among the three groups of patients (*P* > 0.05), indicating comparability. The specific data are shown in Table 1.

**Table 1:** Comparison of general data of the three groups of patients.

**Group**	**Number of cases**	**Age (years)**	**Gender (male/female)**	**Disease course (days)**	**Baseline NIHSS score**	**Baseline Barthel Index**
A group	40	55.61 ± 5.22	25/15	14.25 ± 1.53	15.63 ± 3.24	35.61 ± 8.53
B group	40	56.34 ± 4.83	27/13	13.84 ± 1.35	15.82 ± 3.05	36.14 ± 8.24
C group	40	55.92 ± 5.03	26/14	14.33 ± 1.44	15.72 ± 3.15	35.95 ± 8.35
χ^2^/*F-*value		2.355	2.561	2.179	2.584	2.757
*P-*value		0.876	0.923	0.765	0.954	0.912

### Comparison of NIHSS scores of the three groups of patients at different time points

There was no statistically significant difference in NIHSS scores among the three groups of patients before treatment (*P* > 0.05). Compared with before treatment, the NIHSS scores of the three groups of patients were significantly decreased at 4, 8, and 12 weeks after treatment (*P* < 0.05). At 4, 8, and 12 weeks after treatment, the NIHSS scores of the acupuncture combined with rehabilitation training group were lower than those of the rehabilitation training group and the control group, and the NIHSS scores of the rehabilitation training group were lower than those of the control group, with statistically significant differences (*P* < 0.05). The specific data are shown in Table 2.

**Table 2:** Comparison of NIHSS scores of the three groups of patients at different time points.

**Group**	**Number of cases**	**Before treatment**	**4 weeks after treatment**	**8 weeks after treatment**	**12 weeks after treatment**
A group	40	15.63 ± 3.24	12.55 ± 2.84	10.25 ± 2.52	8.52 ± 2.04
B group	40	15.86 ± 3.05	11.03 ± 2.56	8.83 ± 2.24	7.05 ± 1.81
C group	40	15.74 ± 3.12	9.55 ± 2.03	7.23 ± 1.86	5.5 ± 1.55
*F*-value (between groups)		0.125	10.678	8.923	12.345
*P*-value (between groups)		0.884	< 0.001	< 0.001	< 0.001
*F*-value (within groups)			45.658	48.365	49.347
*P*-value (within groups)			< 0.001	< 0.001	< 0.001

### Comparison of Barthel Index scores of the three groups of patients at different time points

There was no statistically significant difference in Barthel Index scores among the three groups of patients before treatment (*P* > 0.05). Compared with before treatment, the Barthel Index scores of the three groups of patients were significantly increased at 4, 8, and 12 weeks after treatment (*P* < 0.05). At 4, 8, and 12 weeks after treatment, the Barthel Index scores of the acupuncture combined with rehabilitation training group were higher than those of the rehabilitation training group and the control group, and the Barthel Index scores of the rehabilitation training group were higher than those of the control group, with statistically significant differences (*P* < 0.05). The specific data are shown in Table 3.

**Table 3:** Comparison of Barthel Index scores of the three groups of patients at different time points.

**Group**	**Number of cases**	**Before treatment**	**4 weeks after treatment**	**8 weeks after treatment**	**12 weeks after treatment**
A group	40	35.63 ± 8.54	42.51 ± 9.04	50.23 ± 10.05	58.55 ± 10.53
B group	40	36.16 ± 8.22	48.04 ± 9.52	56.83 ± 10.53	65.03 ± 11.06
C group	40	35.93 ± 8.34	55.52 ± 10.05	65.26 ± 11.02	75.54 ± 12.01
*F*-value (between groups)		0.954	6.789	9.876	14.567
*P*-value (between groups)		0.398	0.002	< 0.001	< 0.001
*F*-value (within groups)			16.789	19.876	22.345
*P*-value (within groups)			< 0.001	< 0.001	< 0.001

### Comparison of serological indicators before and after treatment among three groups of patients

Before treatment, there were no statistically significant differences in the levels of serum BDNF, NGF, IL-6, and TNF-α among the three groups of patients (*P* > 0.05). Twelve weeks after treatment, compared with the control group, the levels of serum BDNF and NGF in the rehabilitation training group and the acupuncture combined with rehabilitation training group increased significantly, while the levels of IL-6 and TNF-α decreased significantly (*P* < 0.05). Compared with the rehabilitation training group, the levels of serum BDNF and NGF in the acupuncture combined with rehabilitation training group increased more significantly, and the levels of IL-6 and TNF-α decreased more significantly, with statistically significant differences (*P* < 0.05). The specific data are shown in Table 4.

**Table 4:** Comparison of serological indicators before and after treatment among three groups of patients.

**Group**	**Number of cases**	**BDNF (pg/mL) before treatment**	**BDNF (pg/mL) after treatment**	**NGF (pg/mL) before treatment**	**NGF (pg/mL) after treatment**	**IL-6 (pg/mL) before treatment**	**IL-6 (pg/mL) after treatment**	**TNF-α (pg/mL) before treatment**	**TNF-α (pg/mL) after treatment**
Group A	40	15.62 ± 3.23	20.54 ± 4.04	18.52 ± 3.55	22.02 ± 4.03	25.63 ± 5.04	22.05 ± 4.56	30.51 ± 6.03	27.02 ± 5.52
Group B	40	15.83 ± 3.02	25.0 4± 4.51	18.81 ± 3.33	26.54 ± 4.53	25.8 4± 4.85	18.5 4± 4.03	30.85 ± 5.83	23.50 ± 5.02
Group C	40	15.70 ± 3.14	30.55 ± 5.02	19.0 3± 3.46	32.0 5± 5.02	25.73 ± 4.91	15.02 ± 3.54	30.74 ± 5.93	20.02 ± 4.53
*F*-value (between groups)		0.923	7.890	0.876	8.923	0.954	6.789	0.987	7.890
*P*-value (between groups)		0.402	0.001	0.423	0.000	0.398	0.002	0.387	0.001

## Discussion

### Mechanistic pathways linking key evaluation metrics to stroke recovery

The observed improvements in neurological function (NIHSS scores) and activities of daily living (Barthel Index) in this study are underpinned by synergistic mechanisms involving neuroplasticity, neurotrophic modulation, and anti-inflammatory effects. These pathways align with both traditional Chinese medicine (TCM) principles and modern molecular biology, providing a comprehensive framework to explain the efficacy of acupuncture combined with rehabilitation training.

#### Neuroplasticity and neurological function recovery (NIHSS)

Neuroplasticity, the brain's adaptive reorganization of neural networks post-stroke, is central to functional recovery (11, 12). The significant reduction in NIHSS scores in Group C (acupuncture + rehabilitation) reflects enhanced neuroplasticity, driven by dual interventions:

##### Acupuncture

According to TCM theory, stimulating acupoints such as Jianyu (LI15) and Quchi (LI11) regulates qi (vital energy) and xue (blood) circulation, resolving stagnation and nourishing brain tissues. Modern studies confirm that acupuncture activates mechanoreceptors, triggering sensory afferent signals that enhance cortical excitability and motor map reorganization in the primary motor cortex (M1) (13, 14). For instance, electroacupuncture at LI15 increases regional cerebral blood flow (rCBF) and upregulates PI3K/Akt signaling, promoting neuronal survival and axonal sprouting (15, 16).

##### Rehabilitation training

Task-specific exercises (e.g., Bobath techniques) reinforce synaptic plasticity through repetitive motor learning, inducing long-term potentiation (LTP) in corticospinal pathways (17). This aligns with the TCM concept of “re-establishing harmony” between body and mind through guided movement.

The synergistic effect of acupuncture and rehabilitation is evidenced by elevated serum BDNF and NGF levels in Group C (Table 4). BDNF, a key mediator of synaptic plasticity, enhances dendritic arborization via TrkB receptor activation (18), while NGF supports cholinergic neuron integrity, critical for motor-cognitive integration (19, 20). These neurotrophic factors are upregulated through acupuncture-induced PI3K/Akt pathway activation and rehabilitation-driven glutamate release, collectively reducing neurological deficits (21, 22).

#### Barthel index and functional reintegration: bridging motor recovery and daily adaptation

The Barthel Index improvements in Group C correlate with restored motor coordination and adaptive learning, mediated by both central and peripheral mechanisms:

##### Acupuncture

Stimulating Sanyinjiao (SP6) and Zusanli (ST36) modulates spinal reflex circuits and supraspinal pathways, improving muscle tone and gait symmetry (23). TCM attributes this to balancing Yin-Yang and harmonizing the Liver and Spleen meridians, which govern limb movement.

##### Rehabilitation training

Balance and ADL training enhance proprioceptive feedback and motor planning via cerebellar-thalamocortical loops. For example, weight-bearing gait training activates the supplementary motor area (SMA), facilitating motor relearning (24).

The greater BDNF elevation in Group C (30.55 ± 5.02 pg/mL vs. 25.04 ± 4.51 pg/mL in Group B, *P* < 0.05) further supports motor recovery. BDNF enhances synaptic efficiency in the motor cortex, directly translating to improved ADL performance.

#### Anti-inflammatory and neuroprotective mechanisms

The intervention's anti-inflammatory effects are critical for mitigating secondary brain injury. Group C exhibited the most pronounced reductions in IL-6 (15.02 ± 3.54 pg/mL) and TNF-α (20.02 ± 4.53 pg/mL), aligning with two mechanisms:

##### Acupuncture

By downregulating NF-κB signaling, acupuncture reduces pro-inflammatory cytokine release (e.g., IL-6, TNF-α) and inhibits microglial activation (25, 26). TCM interprets this as “clearing heat and detoxifying” through meridian regulation.

##### Rehabilitation training

Physical activity modulates systemic immune responses, increasing anti-inflammatory cytokines (e.g., IL-10) and reducing oxidative stress.

These effects synergistically preserve peri-infarct neurons and create a permissive microenvironment for neural repair, as evidenced by NIHSS and Barthel Index improvements (Tables 2, 3).

#### Mapping findings to established pathways

BDNF/NGF Upregulation → Neuroplasticity: Elevated BDNF (30.55 pg/mL) and NGF (32.05 pg/mL) in Group C (Table 4) correlate with PI3K/Akt activation, promoting axonal regeneration and synaptic plasticity (27).

- IL-6/TNF-α Reduction → Anti-Inflammation: Suppressed NF-κB signaling reduces neuroinflammation, preserving blood-brain barrier integrity and neuronal survival (28).
- Cortico-Spinal Integration: Acupuncture enhances M1 excitability, while rehabilitation strengthens corticospinal tract connectivity, enabling functional motor recovery (29, 30).

##### Integrating TCM theory with modern mechanisms

TCM attributes stroke recovery to restoring qi-xue balance and unblocking meridians. Acupuncture at LI15 and GB34 activates the Yangming and Shaoyang meridians, which govern limb function, aligning with modern evidence of PI3K/Akt-mediated neuroprotection. Rehabilitation training complements this by “invigorating the sinews” (Jin), enhancing physical resilience through repetitive motion.

## Limitations and future directions

To address the limitation of insufficient innovation in the current study design and further clarify the mechanistic basis of acupuncture combined with rehabilitation training in improving neurological outcomes, future research will incorporate advanced molecular biology techniques and neuroimaging modalities.

In terms of molecular biology techniques, gene and protein expression analysis will be a key focus. Quantitative real—time polymerase chain reaction (qRT—PCR) will be employed to measure the expression levels of genes related to neuroplasticity, such as those encoding synaptic proteins and components of the PI3K/Akt signaling pathway. Western blot analysis will be used to detect the corresponding protein levels, providing a more comprehensive understanding of the molecular changes at the translational level. Additionally, immunohistochemistry will be utilized to visualize the spatial distribution of these molecules in brain tissue, helping to identify the specific regions where the treatment—induced changes occur. This approach will enable us to directly observe how acupuncture combined with rehabilitation training modulates the molecular machinery underlying neural repair and functional recovery.

Regarding neuroimaging modalities, functional magnetic resonance imaging (fMRI) and diffusion tensor imaging (DTI) will play crucial roles. fMRI will be used to monitor brain activity patterns during specific motor tasks or at rest, allowing us to observe in real—time how the combined treatment affects neural activation in different brain regions involved in motor control, sensory processing, and cognitive function. It can reveal whether the treatment promotes the recruitment of alternative neural pathways or enhances the connectivity within existing ones. DTI, on the other hand, will provide valuable information about the integrity and orientation of white matter tracts. By analyzing changes in fractional anisotropy and mean diffusivity values over time, we can assess the extent of axonal damage and regeneration, as well as the improvement in neural communication pathways. The integration of these neuroimaging techniques with molecular biology analysis will offer a more complete picture of the structural and functional changes in the brain, thereby providing a more in—depth understanding of the mechanisms by which acupuncture combined with rehabilitation training exerts its therapeutic effects on stroke patients.

## Data Availability

The raw data supporting the conclusions of this article will be made available by the authors, without undue reservation.
